# Chronic Kidney Disease: Early Detection, Mechanisms, and Therapeutic Implications

**DOI:** 10.3390/jpm13101447

**Published:** 2023-09-28

**Authors:** Charlotte Delrue, Marijn M. Speeckaert

**Affiliations:** 1 Department of Nephrology, Ghent University Hospital, 9000 Ghent, Belgium; charlotte.delrue@ugent.be; 2 Research Foundation-Flanders (FWO), 1000 Brussels, Belgium

Chronic Kidney Disease (CKD) constitutes a global health crisis, silently affecting millions worldwide. This condition, characterized by a gradual decline in kidney function, places a significant burden on healthcare systems and profoundly affects an individual’s quality of life. In this editorial from the Special Issue “Chronic Kidney Disease: Early Detection, Mechanisms, and Therapeutic Implications”, we emphasize the importance of early detection, the role of innovative diagnostic tools, and the ongoing research efforts in the battle against CKD. CKD progresses slowly, often remaining asymptomatic until its advanced stages. Despite its widespread implications, our ability to promptly identify and manage this clinical condition has been hindered by a lack of early, predictive, and non-invasive biomarkers. Currently, kidney function is assessed using diverse equations to analyze the levels of serum creatinine, cystatin C, and albuminuria, and the estimated glomerular filtration rate (eGFR). In the paper titled “Emerging Biomarkers for Early Detection of Chronic Kidney Disease”, we delve into the ongoing efforts to unearth novel biomarkers that could enhance early detection and monitoring, with the aim of improving patient outcomes [1]. However, despite pioneering research, we must acknowledge that we have not yet identified a single biomarker that fully meets the criteria for an ideal early CKD marker. The search for superior methods for the early detection of CKD remains an urgent challenge. Although omics analyses show promise, it is imperative to scrutinize their practicality and cost effectiveness. In the interim, a strategic approach featuring risk assessments within patient populations and rigorous preclinical evaluations may offer a viable path forward in the fight against CKD. A second article “Nomogram-Based Chronic Kidney Disease Prediction Model for Type 1 Diabetes Mellitus Patients Using Routine Pathological Data” sheds light on the development of a predictive model that leverages readily available routine checkup data to identify at-risk individuals [2]. Eight features form the bedrock of this prediction model: age, duration of diabetes, hypertension, triglyceride levels, low-density lipoprotein cholesterol levels, smoking and drinking habits, and intake of ACE inhibitors. By employing a multivariate logistic regression model and readily available features, this model achieved a remarkable accuracy of 90.04% in internal validation and an 88.59% accuracy rate in test data validation. What distinguishes this study is its provision of CKD prediction equations and a nomogram that can serve as a secondary decision-support system for healthcare professionals. These tools can be seamlessly integrated into routine checkups for individuals with type 1 diabetes mellitus, facilitating the early identification of CKD and heightening the overall quality of healthcare dispensed to this vulnerable group.

Innovative technologies, such as Raman spectroscopy, have emerged as promising assets for the diagnosis of kidney diseases. Delrue et al. highlight the potential of this non-invasive technique in early CKD detection and monitoring [3]. Its ability to furnish a molecular fingerprint of kidney diseases and unveil metabolomic changes seamlessly aligns with the latest advances in nephrology. However, Raman spectroscopy data must be rigorously compared with the high standards that are currently employed in nephrology. This comprehensive approach is indispensable to validate the utility of Raman spectroscopy in clinical practice. Raman spectroscopy holds significant potential as a non-invasive and highly informative tool that can contribute to earlier detection and a more effective management of CKD, thereby advancing our efforts against this global epidemic.

While no non-invasive CKD biomarkers currently exist, recent works have been focused on small RNAs within urinary exosomes for the assessment of kidney function. Huang et al. demonstrated significant progress by isolating human urinary exosomes using a combination of ultracentrifugation and SEC column purification, addressing contamination concerns [4]. The choice of isolation method should align with the research goals. For targeting a few small RNAs, conventional ultracentrifugation may suffice. However, comprehensive studies seeking numerous diagnostic biomarkers should consider ultracentrifugation followed by SEC column purification. Identifying specific exosomal biomarkers or small RNA clusters within intact human urinary exosomes holds the potential to complement traditional renal function tests, advancing CKD research toward early detection and more effective management.

CKD, marked by uremic toxin accumulation impacting multiple organs, involves complex factors influencing protein-bound uremic toxin (PBUT) levels, notably, free plasma concentrations. In a 5.5-year prospective study of 523 non-dialysis CKD patients across G1 to G5 stages [5], findings showed a negative correlation between albumin and hemoglobin levels, and both total and free p-cresyl sulfate (pCS) and p-cresyl glucuronide (pCG) concentrations. Multiple linear regression analyses unveiled associations between PBUTs and various factors, including negative links with eGFR and hemoglobin levels. Serum albumin exhibited a distinct pattern, and a multivariate Cox regression analysis highlighted its independent predictive value for adverse outcomes, separate from pCS and pCG associations. Notably, no significant albumin interactions with uremic toxins were observed. This suggests that lower pCS and pCG concentrations correlate with higher serum albumin and hemoglobin levels, hinting at two potential blood pathways mitigating PBUT vasculotoxic effects. Crucially, cardiovascular risk associated with PBUTs extends beyond albumin levels, which remain robust predictors of adverse outcomes. Some uremic toxins originate from the bacterial metabolism of aromatic amino acids in the colon, prompting a closer examination of the relationship between the gut microbial composition and CKD progression. In a cross-sectional study, Gryp et al. revealed that there are no gradual differences in gut microbial composition across different CKD stages [6]. However, a decrease in the abundance of the *Butyricicoccus* genus with worsening kidney function underscores the importance of delving deeper into the functional aspects of the gut microbiome in patients with CKD. Further investigations accounting for the functional capacity of the gut microbiome are imperative to identify potential therapeutic targets for alleviating chronic inflammation and mitigating the impact of uremic toxins in CKD.

Lifestyle choices and habits have a substantial influence on the development of CKD. The paper “Early Diagnosis of Kidney Damage Associated with Tobacco Use: Preventive Application” accentuates a direct relationship between smoking and early subclinical kidney damage [7]. Significantly, the study has identified a panel of biomarkers capable of detecting this condition, including Neutrophil gelatinase-associated lipocalin, Kidney injury molecule-1, *N*-acetyl-beta-D-glucosaminidase, Transferrin, and Ganglioside-activating protein GM2. Crucially, this research has also underscored that subclinical damage persists in individuals who continue to smoke, but can be reversed upon smoking cessation. By identifying subclinical kidney damage and providing a panel of biomarkers for early detection, this study marks a pioneering step in the prevention of smoking-related CKD and the avoidance of acute events stemming from potentially nephrotoxic treatments in smokers.

Finally, children with CKD face unique challenges. The paper “Hydrogen Sulfide-to-Thiosulfate Ratio Associated with Blood Pressure Abnormalities in Pediatric CKD” explores the connection between biomarkers and blood pressure issues in these young patients [8]. Identifying children at high risk for cardiovascular disease (CVD) and providing timely treatment can prevent future CVD events and mortality. Hydrogen sulfide (H_2_S) plays a role in both CVD and CKD. Thiosulfate, a H_2_S derivative, has also been studied. Researchers found that H_2_S, Thiosulfate, and their ratio correlated differently with CVD risk markers in 56 children and adolescents with CKD stages G1–G4. Some children with CKD exhibit higher blood pressure, even in early stages. Patients with abnormal blood pressure had distinct H_2_S-to-Thiosulfate ratios. These findings suggest potential strategies for combating CVD in young patients with CKD by targeting the H_2_S signaling pathway. Further research is necessary, especially for early stage CKD. Understanding these biomarkers can refine risk assessments and personalized CKD care. Exploring H_2_S may open the door for novel CVD therapies for pediatric CKD.

This editorial underscores the importance of early detection, innovative diagnostics, and dedicated research in the battle against CKD. While the search for an ideal non-invasive biomarker continues, progress is ongoing. Risk assessments and rigorous preclinical evaluations are capable of guiding us toward early CKD detection. Key highlights in CKD research include nomogram-based predictive models, such as those for patients with type 1 diabetes mellitus, potentially enabling the early detection of CKD using routine data, leading to improved patient care. Innovative technologies, such as Raman spectroscopy, have potential as non-invasive tools, pending rigorous validation. Urinary exosome research promises specific CKD risk biomarkers, thereby advancing early detection. The direct link between smoking and subclinical kidney damage emphasizes the importance of early detection and biomarkers. Investigating the role of the gut microbiome in CKD progression has uncovered therapeutic targets. Pediatric CKD research, including that on the H_2_S-to-Thiosulfate ratio, offers pathways to reduce cardiovascular risks. Our unwavering commitment to understanding CKD, innovative diagnostics, and personalized care increases global healthcare quality.
